# Editorial: Sustainable nutritional strategies for improving health status, egg and meat quality in poultry

**DOI:** 10.3389/fvets.2026.1828546

**Published:** 2026-05-07

**Authors:** Daniel Mierlita, Adrian Maximilian Macri

**Affiliations:** 1 Department of Animal Nutrition, University of Oradea, Oradea, Romania; 2 Department of Animal Nutrition, University of Agricultural Sciences and Veterinary Medicine, Cluj-Napoca, Romania

**Keywords:** food safety, health, microbiota intestinal, phytochemicals, probiotics, productivity, protein feed

## Introduction

Ensuring animal health and providing safe and affordable foods of animal origin that can meet the growing global demand, while respecting the three pillars of sustainability (environmental, economic, and consumer dimensions), represents a major, complex, and multifactorial challenge. In this context, the present Research Topic explores a range of sustainable nutritional strategies that may help maintain a balance between productivity, poultry health, egg and meat quality, human food security, and environmental sustainability. Particular attention has been given to the ways in which nutritional strategies can influence not only intestinal health and production performance but also the overall health status of poultry, including immune responses and antioxidant capacity. Understanding these interactions is essential for developing feeding approaches that support both productive efficiency and animal welfare. The aim of this Research Topic is to bring together studies that contribute to improving poultry nutrition and feeding practices, with the goal of enhancing productivity, quality, and safety of animal-derived products, while also supporting poultry health and welfare. At the same time, these contributions address broader sustainability goals in modern livestock production systems, including the application of circular economy principles.

Overall, these efforts aim to promote sustainable nutritional strategies that maintain a balance between productivity, poultry welfare, and consumer safety, while integrating metabolomics as an important bridge between genotype and phenotype in the development of precision nutrition approaches.

The Research Topic “*Sustainable Nutritional Strategies for Improving Health Status, Egg and Meat Quality in Poultry*” brings together a collection of 26 articles, including both original research and review papers, that present recent advances and innovative perspectives in poultry nutrition. The contributions address several current and relevant topics through a multidisciplinary and transdisciplinary approach, such as innovative feed additives and ingredients, productivity and feed efficiency, gut microbiota and immune function, metabolism, serum biochemistry and antioxidant capacity, intestinal health and welfare, as well as the safety and quality of poultry products. In total, the collection includes 24 original research articles and 2 review papers authored by researchers from different countries.

The studies included in this Research Topic mainly focus on evaluating the effects of various sustainable nutritional strategies on productive performance, the nutritional and functional quality of poultry products (meat and eggs), serum biochemical parameters, immune responses, antioxidant activity, and the balance of the intestinal microbiome. Another important aspect addressed in this collection is the use of unconventional feed resources rich in nutrients and bioactive compounds. Such resources may contribute to improving the sustainability of poultry production systems, enhancing both intestinal and overall poultry health, and supporting the production of animal-derived foods (meat and eggs) with improved nutritional profiles that are safe for consumers.

## Plant-derived nutritional interventions and functional feed additives

Poultry meat has become one of the most important sources of animal protein in the human diet, largely due to the high yield and production efficiency of broiler chickens, achieved through intensive genetic selection and continuous improvements in nutritional programs. However, modern broiler production practices may negatively affect meat quality as a result of several factors, including high stocking density and rapid growth rate, digestive disorders associated with bacterial and parasitic infections, heat stress, and the contamination of feed with mycotoxins and oxidized oils (Abd El-Hack et al., Oni and Oke).

Research on broiler nutrition increasingly focuses on evaluating the effects of unconventional feed ingredients and bioactive compounds such as phytogenic substances, enzymes, probiotics, and prebiotics. Studies have shown that modulating the nutritional composition of diets and supplementing them with bioactive compounds can positively influence growth performance, meat quality, body composition of broiler chickens, gut microbiota composition and intestinal health, immune function, and nutrient absorption (Gao et al.; Shams-Eldin et al.).

Plant-derived bioactive ingredients and plant extracts show considerable potential as natural feed additives that may serve as alternatives to antibiotics (1).

Dietary supplementation of broiler chickens with polyphenols and flavonoids extracted from *Piper aduncum* represents a promising nutritional strategy and a sustainable alternative to antibiotic growth promoters. Such supplementation has been shown to improve intestinal health, stimulate immune responses, and enhance performance indicators and meat quality by modulating the gut microbiota and intestinal villus morphology (Paredes-Lopez et al.). Similar effects have been reported following dietary supplementation with extracts from areca nut (*Areca catechu* L.), further supporting the potential of this phytogenic compound as a novel feed additive in poultry nutrition (Xu J. et al.).

Another study conducted by Ding et al. demonstrated the beneficial effects of Chinese medicinal plants co-fermented with bacteria and enzymes on growth performance, apparent nutrient digestibility, meat quality, and immune function in broiler chickens. Similarly, dietary supplementation with a powder derived from four Chinese medicinal plant species (1,500 mg/kg) improved growth performance and intestinal health in broiler chickens through multiple mechanisms. These included the maintenance of cecal microbial homeostasis, modulation of lipid and amino acid metabolism, and the potential involvement of the peroxisome proliferator-activated receptor (PPAR) signaling pathway, as suggested by bioinformatics and gene expression analyses (Xu H. et al.).

An effective strategy for improving zootechnical performance in broiler chickens under heat stress conditions is dietary supplementation with a plant-derived natural product (Fibrafid), which has been shown to enhance meat quality and intestinal integrity, even in diets with reduced nutrient density, compared with a commercial prebiotic (Al-Garadi et al.). In the same context, the results reported by Al-Marzooqi et al. demonstrated that dietary supplementation with Alterion (a commercial probiotic) modulates the diversity and composition of intestinal microbial communities. Specifically, it increases bacterial diversity without disrupting the ecological balance of the gut microbiota in both indigenous and commercial chicken breeds raised under high ambient temperatures. This study provides novel insights and further supports the benefits of probiotic supplementation for slow-growing broiler chickens, whose physiology and responses to feed additives may differ significantly from those of commercial lines.

An innovative approach involves combining probiotics with plant extracts—such as an extract derived from the root of *Glycyrrhiza uralensis*—in broiler diets. This strategy has shown positive synergistic effects, leading to improved immune function, enhanced nutrient metabolism, increased antioxidant capacity, and better production performance compared with the separate use of these additives (Lu et al.).

In recent years, growing interest has been directed toward the use of *Bacillus spp*. as probiotics in poultry diets (2); however, evidence supporting the use of *Paenibacillus polymyxa* has been limited. In this context, the studies conducted by Reda et al. demonstrated significant benefits of supplementing the diet of Japanese quails with a probiotic mixture consisting of *Bacillus coagulans* and *Paenibacillus polymyxa* (Bc+Pp), resulting in improved growth performance and feed conversion ratio. The results further indicate improvements in antioxidant and immune status, optimization of digestive enzyme activity, and a favorable modulation of the cecal microbiota through increased populations of lactic acid bacteria and reduced levels of pathogens such as *Salmonella* and *E. coli*.

High-fat diets are widely used in the poultry industry; however, they may negatively affect carcass quality by promoting excessive abdominal fat deposition (3). To improve meat quality and reduce abdominal fat accumulation, Liu et al. supplemented broiler diets with exogenous bile acids (BA), which significantly reduced the mRNA expression of genes associated with lipogenesis while increasing the expression of genes related to lipolysis in the liver. In addition, BA supplementation improved production performance, serum enzyme activity, lipid metabolism, and intestinal morphology in broiler chickens, thereby providing a theoretical basis for the application of bile acids in broiler production systems.

## Innovative approaches for optimizing diet composition and modulating metabolism

The implementation of targeted nutritional modifications represents a viable strategy for optimizing food safety while maintaining poultry production performance. Because the consumption of poultry meat contaminated with *Campylobacter jejuni* represents a major route for the transmission of zoonotic pathogens to humans, controlling the intestinal microbiota at the farm level is essential for reducing risks along the food chain. In this context, dietary manipulation—through reducing dietary iron and phosphorus levels while supplementing phytase to meet mineral requirements—has demonstrated considerable potential for limiting pathogen colonization. The study conducted by Gibbs et al. confirmed that average Campylobacter loads in the cecum were numerically 7.7 times lower in the treatment supplemented with phytase and without added iron and inorganic phosphate.

Untargeted metabolomic analysis revealed that lactulose modulates glycerophospholipid and fatty acid metabolism, mechanisms that play an essential role in maintaining intestinal barrier integrity and the overall health status of geese (Wang et al.). Supplementation of high-protein diets with 0.30% lactulose significantly improved growth performance in geese while simultaneously reducing serum uric acid concentrations. Lactulose exerts a strong prebiotic effect by stimulating the proliferation of beneficial microorganisms and reducing pathogenic aerobic bacteria such as *Escherichia coli* and *Salmonella*, thereby contributing to intestinal homeostasis and improving intestinal morphological architecture.

In response to the increasing emphasis on improving nutrient utilization efficiency and reducing environmental impact, the studies conducted by Jiang et al. demonstrated that probiotic-fermented feed (*Bacillus paralicheniformis* LN33) has strong potential for application in poultry production. This approach represents an innovative strategy for enhancing both the economic efficiency and sustainability of the poultry industry. When incorporated into meat duck diets at a level of 3%, fermented feed improved growth performance, feed conversion ratio, and overall health status by stimulating antioxidant defenses, modulating immune responses, and reshaping the intestinal microbiota.

Dietary supplementation with different metal–amino acid complexes (minerals complexed with amino acids or with lysine and glutamic acid) improved eggshell quality and resistance to Salmonella Enteritidis penetration in table eggs without negatively affecting laying performance (Clemente et al.).

Finally, the study conducted by Peng et al. provides valuable insights into the endocrine mechanisms underlying improved laying performance following dietary supplementation with N-carbamylglutamate, a functional feed additive involved in hepatic lipid metabolism modulation and the upregulation of genes associated with lipid synthesis. Similarly, canthaxanthin may be successfully used to mitigate oxidative stress–induced hepatic lipid metabolism disorders and reduced egg production in poultry (Chen J. et al.).

## Innovative ingredients in poultry nutrition: a sustainable strategy for the circular economy

Conventional poultry diets rely primarily on soybean meal as the main source of protein, as it closely meets the nutritional requirements of poultry. However, there is currently an increasing gap between the production, availability, and demand for soybean products and by-products used in animal feeding. Consequently, identifying and utilizing alternative feed resources has become essential for ensuring adequate feed supply for animal production. The studies included in this Research Topic evaluated the possibility of partially replacing soybean meal with *Scarabaeiform larvae* meal (SL) in slow-growing broiler chickens (Chen S. et al.), or with defatted black soldier fly larvae meal (BSFL) and insect-derived waste from *Periplaneta americana* in laying hens (Chen L. et al.; Tao et al.). The results suggest that SL, BSFL, and insect-derived by-products, due to their favorable nutritional and functional profiles, can effectively substitute part of the soybean meal in diets for slow-growing broilers and laying hens raised in ecological production systems. This approach not only improved growth performance, eggshell thickness, and the overall physiological status of the birds, but also contributes to reducing the pressure on conventional protein resources while providing a scientific framework for the efficient utilization of unconventional feed resources within the context of the circular economy.

The identification of high-quality and high-yield alternative feed resources has also been a key objective of studies proposing the use of a perennial plant from the *Urticaceae* family, commonly known as ramie (*Boehmeria nivea*) or “China grass,” in meat duck diets. Replacing the standard diet with fermented ramie mixed with wheat bran (1:1) for a period of 90 days improved the growth performance of ducks, likely due to its beneficial effects on serum biochemistry, antioxidant capacity, intestinal morphology, and the structure of the intestinal microbiota (Lv et al.). These findings suggest that fermented ramie represents an innovative and efficient feed resource that may help reduce the dependence on conventional feed ingredients such as corn and soybean in poultry production.

## Conclusions

The transition toward modern and sustainable poultry production requires the adoption of a multidisciplinary and transdisciplinary approach that integrates traditional nutritional strategies with advanced fields such as metabolomics. Such an approach prioritizes animal welfare and consumer safety while promoting the use of innovative resources within a circular and resilient production system.

Future research in the field of sustainable nutritional strategies for poultry farming should focus on integrating “Omics” technologies for precision nutrition, using metabolic, genomic, and microbiomic biomarkers to formulate tailored diets capable of simultaneously optimizing production performance, health status, and product quality. At the same time, the exploration and validation of sustainable alternative ingredients, including through life cycle assessment analyses, represent a key direction for supporting gut health and immune response, modulating the quality profile of eggs and meat, and reducing environmental impact. Optimizing the nutritional profile of poultry products, particularly by enriching them with polyunsaturated fatty acids, antioxidants, and micronutrients, without compromising oxidative stability and sensory properties, remains a priority objective that will support the sustainable development of the poultry sector.
